# A Review of Strain-Distributed Optical Fiber Sensors for Geohazard Monitoring: An Update

**DOI:** 10.3390/s25206442

**Published:** 2025-10-18

**Authors:** Agnese Coscetta, Ester Catalano, Emilia Damiano, Martina de Cristofaro, Aldo Minardo, Erika Molitierno, Lucio Olivares, Raffaele Vallifuoco, Giovanni Zeni, Luigi Zeni

**Affiliations:** 1 Department of Engineering, University of Campania “Luigi Vanvitelli”, 80131 Aversa, Italy; agnese.coscetta@unicampania.it (A.C.); ester.catalano@unicampania.it (E.C.); emilia.damiano@unicampania.it (E.D.); martina.decristofaro@unicampania.it (M.d.C.); aldo.minardo@unicampania.it (A.M.); lucio.olivares@unicampania.it (L.O.); raffaele.vallifuoco@unicampania.it (R.V.); luigi.zeni@unicampania.it (L.Z.); 2 Istituto per il Rilevamento Elettromagnetico dell’Ambiente (IREA), National Council of Research (CNR), Via Diocleziano 328, 80124 Naples, Italy; zeni.g@irea.cnr.it

**Keywords:** distributed optical fiber sensor, real-time monitoring, early warning systems, geotechnical monitoring, DAS technology

## Abstract

Geohazards pose significant dangers to human safety, infrastructures, and the environment, highlighting the need for advanced monitoring techniques for early damage detection and structure management. The distributed optical fiber sensors (DFOS) are strain, temperature, and vibration monitoring tools characterized by minimal intrusiveness, accuracy, ease of deployment, and the ability to perform measurements with high spatial resolution. Although these sensors rely on well-established measurement techniques, available for over 40 years, their diffusion within monitoring and early warning systems is still limited, and there is a certain mistrust towards them. In this regard, based on several case studies, the implementation of DFOS for early warning of various geotechnical hazards, such as landslides, earthquakes and subsidence, is discussed, providing a comparative analysis of the typical advantages and limitations of the different systems. The results show that real-time monitoring systems based on well-established distributed fiber-optic sensing techniques are now mature enough to enable reliable and long-term geotechnical applications, identifying a market segment that is only minimally saturated by using other monitoring techniques. More challenging remains the application of the technique for vibration detection that still requires improved interrogation technologies and standardized practices before it can be used in large-scale, real-time early warning systems.

## 1. Introduction

Geohazards can seriously compromise the integrity of urbanized areas, causing damage to the structures and infrastructures, potential fatalities and, in general, relevant losses to communities. Due to their complex and often unpredictable nature, mitigation of these risks often involve a combination of strategies, including improved land-use planning, adoption of early warning systems (EWSs), and, in areas affected by slow or moderate soil ground deformation, monitoring, inspections, and maintenance procedures carried out to ensure proper user well-being and performance of the exposed structures to continue keeping them in use [1,2]. The effectiveness of these monitoring techniques depends on their ability to quickly identify, characterize, and control changes in the performance of the element under observation. This idea is the basis of landslides or earthquake EWS, defined by [3], as the regular and continuous measurement and analysis of key environmental parameters under operating settings to detect any anomalous behaviors in their early stages. In case of an accident or extreme event, such as an earthquake, landslide, or unexpected loadings, EWS can rapidly evaluate and screen the status of the territory, activating emergency procedures to protect lives and structures [4].

EWS for geological hazards, especially for landslides, subsidence, and earthquake detection, needs to include monitoring data showing that an event might occur and predict its magnitude and propagation. They are often based on a network of strategically placed sensors to measure soil movement and acceleration or variation of the factors that indicate the imminent trigger of an event [5]. Traditional devices include inclinometers [6,7], extensometers, geophones, accelerometers, total station surveys, load cells, and GNS-based sensors. While these sensing devices act as single-point sensors, geohazard threats require a widely distributed sensor network to produce repeated and reliable data and monitor large, potentially unstable areas. Furthermore, traditional instruments have various shortcomings, including limited data management capability, a lack of real-time automated monitoring, and interference risk [8].

**Figure 1:**
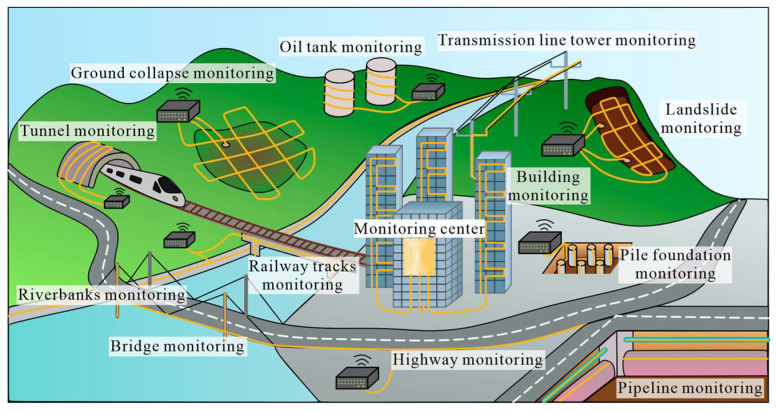
DFOS applications in civil engineering (from Ref. [10]).

These limitations can be overcome using distributed optical fiber sensors (DFOSs), which are gaining increasing interest from the scientific and stakeholder’s communities [9], whose main advantages are long distance monitoring, wide application spectrum (suitable for measuring temperature, humidity, stress, strain, flow velocity, displacement, vibration, radiation, etc.), long life cycle (silica material of optic fiber has long lifespan and is resistant to erosion), high sensitivity and wide dynamic range, high temperature endurance, immunity to electromagnetic interference (used in severe environment), small size, large bandwidth, high-speed transmission, and ease in system integration. Figure 1 shows the potential DFOS applications in civil engineering monitoring, including those related to some geohazards [10].

The main advantage that DFOS monitoring offers over traditional techniques is the vast spatial coverage (unique sensing line from tens of meters to km long) along with real-time processing, which makes it an essential tool in assessing geohazards related to landslides [11,12,13,14,15], subsidences [16,17], and earthquakes [18,19,20]. The continuous data they provide is also crucial for understanding geohazard dynamics. Despite the need for specialized skills in installation and maintenance for fiber-optic systems [11] and the requirement for specific configurations [12,21,22,23], these data can support land use planning and inform mitigation strategies to protect vulnerable areas [24,25,26,27,28].

This article reviews the most recent applications of distributed optical sensing for the most common and hazardous geological phenomena in urbanized areas. The main attention is on those detection techniques capable of measuring soil deformation or vibrations, which provide clear direct indications of the triggering of a hazardous event, discussed based on the field of application, namely for various types of landslides (Section 3.1), for subsidences (Section 3.2), and for earthquakes (Section 3.3). It highlights the prospects and future developments of this distributed monitoring technique, aiming to provide a clear and accessible overview for both specialists and non-expert stakeholders.

## 2. Distributed Optical Fiber Sensors and Techniques

**Figure 2:**
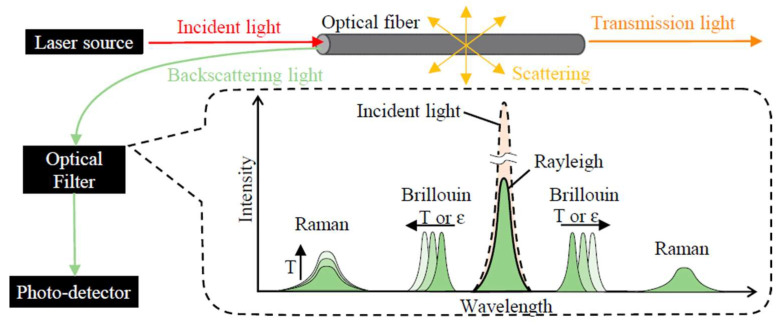
Rayleigh, Raman, and Brillouin scattering in optical fibers (from Ref. [10]).

When propagating through an optical fiber, the light wave is subject to scattering due to different mechanisms. The scattered light can be detected either in the forward or backward direction. As shown in Figure 2, three optical phenomena, Rayleigh, Brillouin, and Raman scattering, can be used for a variety of sensing applications.

Rayleigh scattering is a type of elastic scattering due to fiber inhomogeneities, which means that the frequency of the scattered light remains unchanged. Historically, Rayleigh-scattered light intensity has been used in Optical Time-Domain Reflectometry (OTDR) equipment to determine the location and extent of fiber breakpoints and damage [29,30]. In the last decades, Rayleigh scattering has been widely employed to measure strain, temperature, and vibrations in optical fibers by taking advantage of the influence of these measurands on the relative positions of scatterers along the fiber. Brillouin and Raman scattering are both inelastic scattering phenomena, implying that the frequency of the scattered light shifts during the scattering process [31,32]. In Brillouin scattering, the frequency shift experienced by the scattered light is sensitive to the temperature and strain conditions of the fiber, with the consequence that these measurands can be monitored by tracking the Brillouin frequency shift. In Raman scattering, the intensity of the anti-Stokes component of the scattered light is strongly dependent on temperature, enabling distributed temperature sensing with no strain cross-sensitivity.

In the following discussion, we exclude Raman-based DFOS, as they can detect only temperature changes and therefore are of limited significance in geohazard monitoring. Thus, we summarize in Table 1 the principal DFOS technologies allowing distributed strain measurements, relying on either (backward) Rayleigh or Brillouin scattering. Techniques based on Brillouin scattering are the most studied and used DFOS in the geotechnical engineering field, owing to their extended measurement range capabilities (up to several tens of km). They include Brillouin Optical Time-Domain Reflectometry (BOTDR), Brillouin Optical Time-Domain Analysis (BOTDA), and Brillouin Optical Frequency-Domain Analysis (BOFDA). The BOTDR method has the advantage of requiring single-ended access to the sensing fiber. Besides simplifying fiber deployment, this makes the system “fault-tolerant”, i.e., it can recover the strain distribution even after a single fiber interruption. Methods based on the analysis of the Brillouin gain impressed on a probe light (e.g., BOTDA and BOFDA) ensure a higher signal-to-noise ratio (SNR) and therefore a better accuracy and/or spatial resolution, but require access to both fiber ends and are not fault-tolerant.

Methods based on Rayleigh scattering fall into two categories, i.e., time-domain and frequency-domain schemes. The time-domain methods usually rely on the detection of the phase of the Rayleigh backscatter signal, which is extremely sensitive to any subtle perturbation acting on the fiber. These systems, known as phase-sensitive Optical Time-Domain Reflectometry (phi-OTDR), also provide a very high acquisition rate (in the order of several kHz or more), and therefore are especially suitable for vibration detection. In recent years, Φ-OTDR has been extensively employed to realize the so-called distributed acoustic sensors (DASs), which, also relying on Artificial Intelligence and Machine Learning algorithms, are paving the way to the detection of tiny vibrations originating from rapid events like fast landslides and earthquakes. On the other hand, methods relying on the frequency-domain acquisition of the Rayleigh backscatter (the so-called Optical Frequency-Domain Reflectometry, OFDR) exhibit an extremely fine spatial resolution (in the order of a few mm, typically) but are limited in terms of sensing distance (several tens of meters) and are more susceptible to environmental noise.

**Figure 3:**
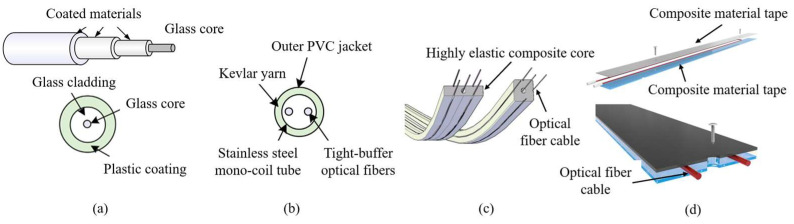
Structure of different optical fiber cables: (**a**) strain sensor fiber cable; (**b**) simple temperature compensation fiber cable; (**c**) 3D monolithic composite strain fiber cable [35]; (**d**) hybrid strain fiber cable NSHT [36].

Most applications in the geoengineering field use dedicated OF sensors for strain and temperature measurements [33]. Depending on the specific application, different fiber cables have been designed and manufactured, from the simplest one based on a tight polymer jacket, to flat cables specially developed for retrieving an independent temperature measurement (Figure 3a,b), or cables in which the fibers are drowned in a monolithic matrix or packaged by composite materials tapes for use in geotechnical and structural fields (Figure 3c,d).

The main task is protecting the sensing core for its use in harsh environments, while providing full strain transfer from the monitored element (soil or structure) to the optical sensor, assuring accurate measurements. However, experimental studies [34,35] highlighted how the presence of a plastic coating, causing interfacial slippage and undergoing viscous deformation over time, affects the optical sensor’s accuracy. The use of monolithic, composite cross-sections with a large elastic range seems to satisfy both the needs of protection and accuracy of the DFOS-based strain sensors [35,36]. Various other types of FO-based sensors for measuring earth pressure, pore pressure, etc., are under development, but this is beyond the scope of this research.

## 3. Geohazards Monitoring

### 3.1. Landslide Monitoring

Landslides are among the most destructive natural phenomena, and the complex interplay between geological, hydrological, and climatic factors that drives these events makes them challenging to study and predict [37]. They include a wide range of slope movements characterized by very different kinematics [38], which require the implementation of specific monitoring tools in EWS. Developments of DFOS systems for landslides are, therefore, discussed based on their kinematics.

#### 3.1.1. Slow Landslide Monitoring

Although traditional geotechnical monitoring of slow landslides includes many types of devices as groundwater pressure transducers, rain-gauges, and moisture probes, the most effective early-warning metric remains the temporal tracking of surface and subsurface movements [39,40], which still suffers from an expensive system of realization and management, and poor spatial and temporal resolution. While GPS and topographic techniques offer precise measurements, they are limited in spatial coverage. Remote sensing technologies like InSAR and LiDAR provide valuable complementary data, with InSAR effective for surface deformation detection and LiDAR excelling in topographical accuracy [41,42,43]. However, these methods focus primarily on superficial kinematics and are limited by latency in data acquisition and processing, especially during sudden landslide activations or reactivations [44].

Information on subsurface movements is, instead, fundamental for understanding the phenomenon and for taking countermeasures. Instruments like inclinometers and extensometers are traditionally used, yet they lack spatial continuity and are challenging to automate [45]. In addition, when the inclinometer tube is subject to large deformation, the probe cannot be lowered deeply into the tube and displacement measurements can no longer be made. Therefore, the research is mainly devoted to the implementation of innovative optical fiber inclinometers [46,47,48,49,50].

**Figure 4:**
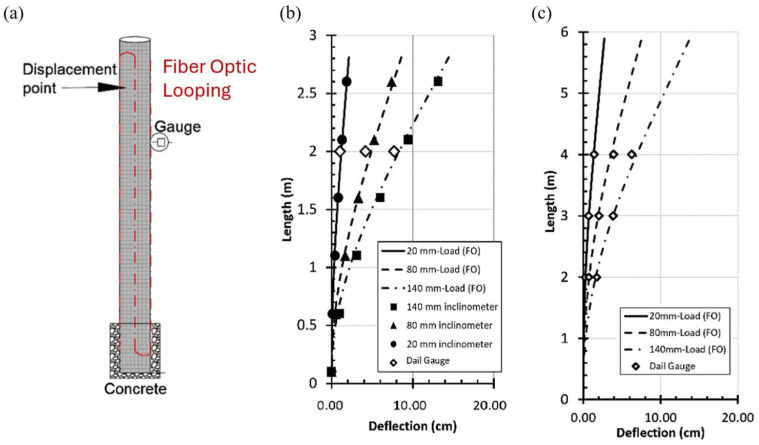
DFOS-inclinometer laboratory setup: (**a**) inclinometer casing test in vertical condition; (**b**) deflection of a 3 m-long ABS inclinometer casing; (**c**) 6 m PVC FO inclinometer [52].

The first attempt to implement a DFOS-based inclinometer dates to the 1990s with a laboratory setup [51]. More recently, Ghazali et al. 2019 [52] validated a fiber-optic inclinometer against traditional systems using a cantilever bending test by attaching a fiber cable along the sides of the inclinometer casing. The BOTDA technique was employed for interrogation, achieving a spatial resolution of 50 cm and a spatial sampling interval of 5 cm. The results demonstrated good agreement between the fiber-optic measurements—recorded every 5 cm—and those of the conventional instrument, which sampled at 50 cm intervals (Figure 4). The test underscores that, despite the two devices sharing the same spatial resolution, the order-of-magnitude difference in spatial sampling (5 cm vs. 50 cm) still yields consistent deflection profiles when strains vary continuously, as observed in bending tests. Different results can be obtained in the case of localized strains as shear slip surfaces in landslides, where the improved spatial resolution of the DFOS-based inclinometer could play a key role.

**Figure 5:**
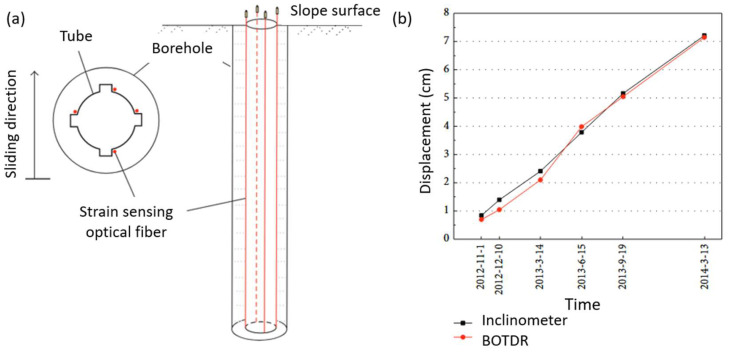
In-field DFOS-inclinometer setup by [52]: (**a**) distribution of sensors in the borehole; (**b**) detected displacements by traditional inclinometer and BOTDR system over time [53].

More recently, many efforts have been made to use this new generation of inclinometers on real slopes, mainly based on BOTDA and BOFDA techniques. Study [53] installed six BOTDR-based fiber-optic inclinometers in the Majiagou landslide. Using the Euler–Bernoulli beam theory, the authors linked strain to slope curvature. Although the system can identify sliding surfaces by revealing increasing localized strain peaks, the operation of strain integration along the length of the pipe leads to displacement errors (~4 mm) of about 10% (Figure 5). In interpreting the strain profiles along the inclinometer tube, [54] proposed the conjugate beam method, enhancing accuracy over Euler–Bernoulli (relative error of 5% against 12%), enabling more precise deflection profile reconstruction. Indeed, the operation of integration of the strain profile for retrieving the displacement profile is not an easy task, mainly due to the assumption made about the integration constants and to the error that propagates. Few tentative studies have been published in this regard, and, among these, [55] obtained a satisfactory agreement between traditional and DFOS-retrieved cumulative displacement profiles by implementing a Machine Learning training procedure, using a BOTDR (Brillouin Optical Time-Domain Reflectometry) fiber-optic interrogation technique with a spatial sampling of 5 cm and an accuracy of 40 micro-strain. The main limit of the application of ML algorithms relies on the fact that they require substantial training samples, leading to increased costs and limited applicability in certain areas.

**Figure 6:**
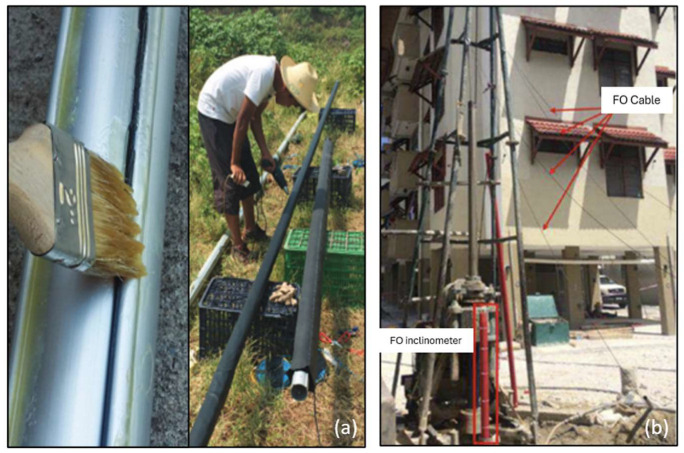
Procedures for installing a DFOS-based inclinometer: (**a**) on-site FO cable gluing along inclinometer tube [53]; (**b**) positioning of pre-equipped tube and finalization of the installation process of FO inclinometer with drilling rig (reprinted from [59] under a CC BY-NC-ND 4.0 license).

Despite these advances, DFOS technology for monitoring internal soil deformation in landslide environments is not yet standardized [56]. Coating materials can influence stress transfer and measurement accuracy, and slippage between coatings and fibers remains an issue [34]. Most inclinometer setups involve gluing optical fiber sensors along the outer surface of a vertical pipe (Figure 6a) or in grooves appropriately made along it; some others require laboratory preparation before on-site installation (Figure 6b). In any case, careful handling to avoid breakage during assembly, especially for long pipe configurations, is required, and the entire operation remains delicate and time-consuming [57,58].

**Figure 7:**
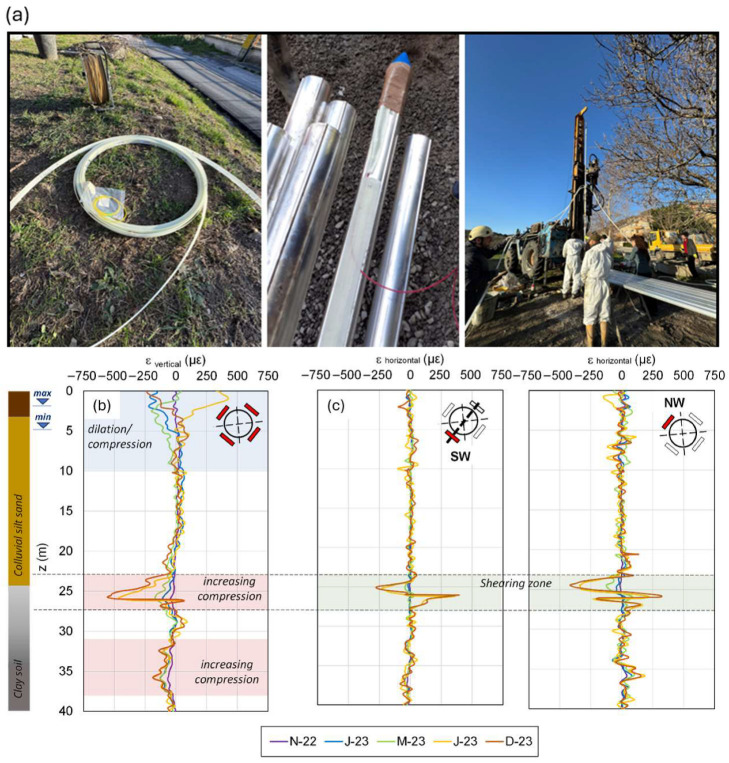
Installation procedure of a Smart Extenso-Inclinometer (**a**), trends of vertical strain (**b**), and shear strain profiles along SW and NW directions (**c**) [44].

A recent effort to address these challenges has been made by [60], who realized a New Smart Hybrid Transducer (NSHT), embedding U-shaped fiber sensors within composite materials. These prefabricated transducers improved stress transfer and mitigated problems during the installation of very long inclinometer pipes. This way, [44,58] realized a Smart Extenso-Inclinometer (SEI) based on the BOTDA technique, which was able to measure both horizontal and vertical strain with a spatial sampling of 5.1 cm and an accuracy of 20 micro-strain and furnish a theoretical interpretation of the data enabling full 3D deformation reconstruction. A field study in Centola, Italy, where a 40 m-long SEI was easily installed by technical workers in 2022 (Figure 7a), demonstrated its ability to distinguish between different movement types, clearly individuating two areas of increasing soil compression (Figure 7b) and a shearing zone at the passage between the colluvial and the basal clayey formations (Figure 7c). The authors also supported integration into LEWS via a strain-based threshold system. The system aims at the reduction of the installation time and costs.

To reduce the cost of the analyzer and the potential damage of the optical fiber during installation, Zheng et al. [61] developed a borehole-installable FOS device based on the OTDR technique embedded in a 36 mm rod and fixed with cement mortar, providing full-length strain measurements. The design allows for potential sensor multiplexing to monitor deep landslide profiles, but it remains a multi-point system more than a distributed one, and further in-field tests are needed to evaluate the effectiveness of this system.

**Figure 8:**
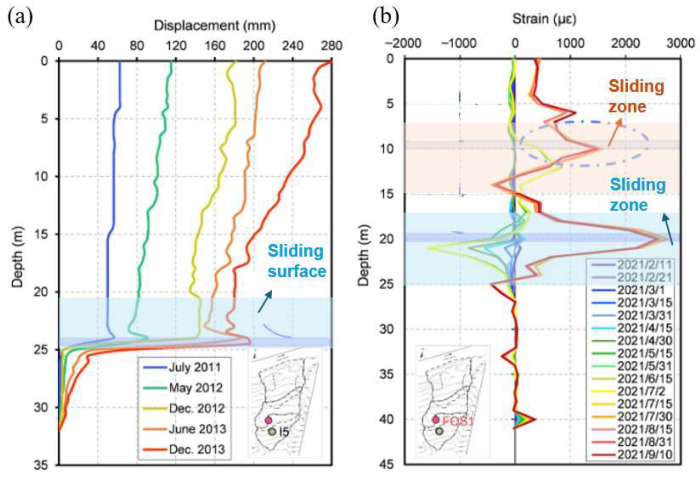
(**a**) Displacement recorded by traditional inclinometer; (**b**) strain distribution detected by WFBG (modified from [62]).

Similarly, Ye et al. [62] used a particular type of fiber grating with ultra-weak reflectivity (WFBG), which allows spatial-temporal near-distributed temperature, moisture, and strain sensing, in the Outang landslide (China), embedding the sensors in a vertical borehole with a diameter of 110 mm to detect deformation and moisture. The results of the WFBG sensors were then compared with those from a traditional inclinometer casing (named I5) (Figure 8a)**.** The WFBG sensors (FOS1 in Figure 8b) detected both a primary sliding surface at about 20 m depth (the different depths at which the main shear surface is identified by the traditional and innovative devices being related to the diverse position of the two devices within the sliding body) and a secondary one at 10 m that traditional inclinometers missed, highlighted in Figure 8b with a dotted circle. Moreover, soil moisture and temperature monitoring allowed investigation of the most effective rain characteristics in accelerating landslide movements. The multi-physical data allows detailed insight into the thermo–hydro–mechanical interactions occurring within the landslide mass beneath the surface. The main shortcoming remains data interpretation complexity, even due to the lack of direct comparison with traditional instruments.

The use of Rayleigh scattering for vertical soil strain measurements in slow landslides is rare due to the limited distance that this technique can cover in the order of tens of meters, but some applications can be found [63,64]. Thanks to the very high strain and spatial resolutions (1.87 με and 10 cm) [64], the measurements provided a clear-cut vertical profile of the strain changes caused by rainfalls that cannot be detected by conventional methods. A main shortcoming remains the cost of the interrogation unit and the limited measurement distance.

DFOS monitoring of surface or near-surface soil movements to the aim of capturing the complex pattern that landslides exhibit has been exploited by [65], who customized strain sensing cables for road-embedded installation in two creeping landslides in Switzerland to locate the boundaries of the sliding masses. Continuous strain was measured by the BOTDA technique for one year, showing a good agreement with visual and geodetic monitoring data. However, the customized cable protection introduced non-linearities in strain-frequency response that required the use of interpretation methods and preliminary calibration laboratory tests, limiting the spreading of this solution.

The potential of using distributed optical fiber sensing (DFOS) technology as a large-scale strain gauge has also been explored by [66]. In their study, sensors were installed both on the surface and within four vertical boreholes at the Majiagou landslide site. The goal was to characterize the deformation occurring at the surface and within the unstable subsurface.

The fiber-optic (FO) sensing line was laid in trenches excavated in the soil to monitor surface deformation. To analyze internal deformation, the fiber-optic cable was also installed in boreholes. Specifically, the cable was attached along the sides of the inclinometer casing, and the Brillouin Optical Time-Domain Reflectometry (BOTDR) technique was used for interrogation. This configuration achieved a spatial resolution of 1 m and an accuracy at the micro-strain level (με).

The authors proposed three geometric models to interpret the different strain distributions observed in optical cables directly inserted into boreholes crossing the slipping zone, reflecting the complex spatial relationship between the sensor cable and the sliding surface. The Orthogonal Model was designed for scenarios in which the cable is approximately perpendicular to the slipping zone, providing a normal type of strain distribution that presents the maximum shear strain at the center of the zone. When the optical cable intersects the slip zone at an angle other than 90° (frequent in the middle and upper parts of the landslide), two Skew Models have been developed. These models differentiated the cable deformation response based on the position of the main sliding surface within the sliding zone (central vs. upper limit), illustrating the evolution of the strain from a compression to a tensile state as the shear displacement increases. The validation of these models, through comparisons with GPS and inclinometer data, underlined the potential of the DFOS for detecting the position of the sliding zone, allowing a better understanding of the evolution of landslides.

Recently, Ouellet et al. (2024) [67] applied DAS technology with nanostrain-rate sensitivity to monitor slow-moving landslides at the Hollin Hill Observatory (UK). By deploying a 925-m fiber-optic cable buried at 10 cm depth, they achieved 4-m spatial and 1-Hz temporal resolution, revealing previously undetected landslide processes—such as strain initiation at the head scarp, rupture zone acceleration, retrogressive deformation, and flow-lobe surges—during a three-day rainfall event. The DAS data have been validated with traditional geotechnical instruments (i.e., ShapeArrays, LiDAR, and GPS data). However, the study emphasizes the need to optimize cable geometry for detecting deeper, multidirectional deformations, positioning DAS as a complementary tool for landslide early-warning systems. Another significant concern is the mechanical coupling between the fiber-optic cable and the surrounding soil. Surface movements, such as flow lobes, can lead to decoupling, rendering strain measurements unreliable, particularly in rapid or shallow failure scenarios.

#### 3.1.2. Fast Landslide Monitoring

In the case of rapid slope movements, which represent a severe geohazard due to their high velocities—often exceeding 100 km/h—and their capacity to mobilize large volumes of soil, rock, and debris within seconds to minutes, research focuses on the integration of DFOS systems within monitoring network, at the aim of providing critical data for the early identification of slope instability. Triggering factors commonly include intense precipitation, seismic events, and anthropogenic modifications of the terrain.

**Figure 9:**
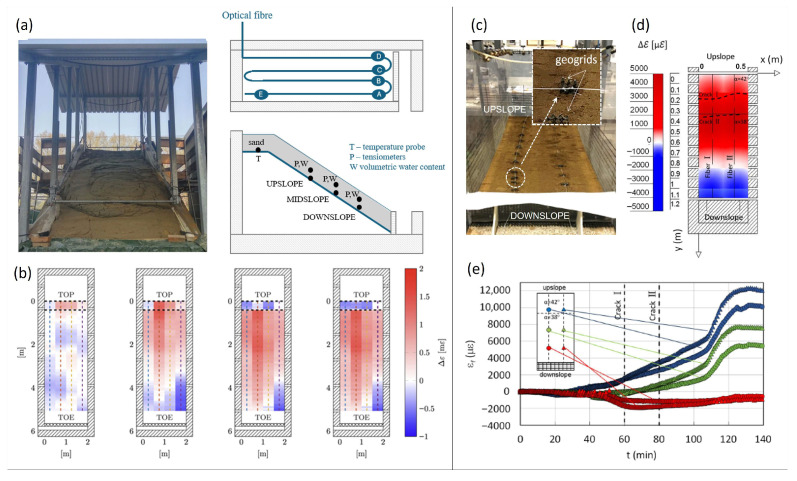
Physical models implementing DFOS for early detection of rapid slope movements: (**a**) model slope and FOS monitoring from [40]; (**b**) strain maps during infiltration test [40]; (**c**) model slope and anchored fiber cables from [71]; (**d**) strain map 80 min after the beginning of rain [71]; (**e**) strain trends at three locations inside the slope [71].

Because of the sudden triggering and the unpredictable detachment area, implementations of DFOS-based monitoring systems at a large scale are very scarce. Physical modeling under controlled conditions was first used to test the effectiveness of DFOS technology for rapid landslide detection [39,40,68,69,70,71]. Fiber cables simply embedded in large- and small-scale sandy slopes subjected to artificial rainfall were tested by [40,71], respectively. In [40], a commercial OFDR unit was used to measure the strain exerted on the cable by the landslide with a spatial resolution of 10 mm (Figure 9a,b), whereas in [71], both BOFDA and BOTDA techniques were employed with spatial resolutions of 2 cm and 50 cm, respectively (Figure 9c–e). In both experiments, the DFOS can follow the soil strain evolution, identifying three phases of landslide development, from progressive soil saturation to volumetric collapse up to the detection of early signs of failure. However, when approaching failure, the slippage between the DFOS and the surrounding soil may hide the detection of imminent slope instability. To counteract the relative displacement between DFOS and soil in the presence of a high-strain field, ref. [71] anchored the fiber cable using small geogrids (Figure 9c). This way, the ability of the DFOS system to detect slope failure was enhanced by recording a strain value ten times higher than those recorded in previous tests (Figure 9e).

**Figure 10:**
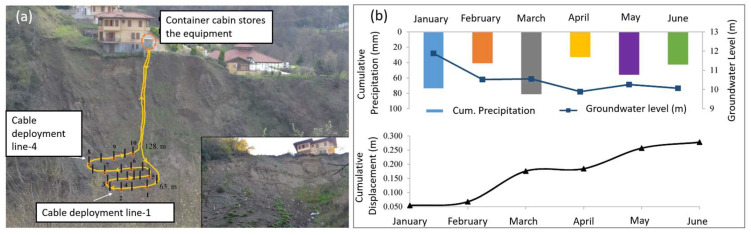
(**a**) Field setup. (**b**) Monthly trends of cumulative precipitation and mean groundwater and cumulative displacement recorded in the monitored period (January–June 2016) (reprinted from [23] under a CC BY 4.0 license).

Moving towards full-scale tests, [23] implemented a monitoring system based on the BOTDA technique in the Bahçecik region in Turkey, an area with a high vulnerability to hydrogeological disturbances. A FOS cable was buried at a depth of 15–20 cm along a cover deposit in a tectonically deformed and disintegrated unit of alternating sandstone–siltstone layers subject to weathering. The FOS was installed according to a layout covering the most critical area (Figure 10), preliminarily identified through geotechnical analyses. To stabilize the installation and ensure stress transfer, the cable was anchored to 21 stakes distributed along the slope around which it was wrapped. All the cables converged into a container located in a stable area, which housed the BOTDA system, a data acquisition unit with remote transmission, and a computer for real-time processing (Figure 10a).

The six-month monitoring campaign enabled a correlation between the evolution of cumulative displacements estimated from measured values of soil strain and environmental variables, including precipitation, groundwater level, and temperature. As reported in Figure 10b, the estimated cumulative displacements increased after the wetter months, highlighting a clear relationship between strain accumulation and meteorological events. The maximum cumulative displacement reached was 0.28 m against 0.25 m measured by surface topography measurements, testifying the reliability of the system in this real-scale application and its potential use in EWS.

#### 3.1.3. Rockfall Monitoring

Mainly for rockfall detection, monitoring of vibration induced by the opening of rock fractures or by rock-mass detachment and propagation is realized via the Φ-OTDR technique applied as a new frontier in geohazards’ EWS. Indeed, when very fast slope events such as snow avalanches and rock falls (sub-second time scale) occur, the traditional DFOS are not useful because they allow efficient monitoring over km-long distances only on a minute time scale. In these cases, distributed acoustic sensing has been tested as an alternative. Indeed, both phenomena are among the most destructive large-scale mass movements, posing serious threats to structures and infrastructure in mountain areas, invading connecting roads, and involving vehicles. Since such phenomena produce detectable seismic signals at the onset of instability, traditional punctual seismic stations have been used, but they often fail to detect weak signals from small events and are almost always lacking real-time data transmission. For this reason, DAS technology has emerged as a promising seismic monitoring approach, able to convert kilometers of optical fiber into a highly sensitive virtual array of acoustic sensors [72]. Moreover, many works also exploit the effectiveness of “dark fibers”, i.e., unused fiber optic in telecommunication cables already installed in road surfaces, for real-time monitoring. Study [73] implemented this system in northern Norway, in an area known for the frequent occurrence of avalanches; between 2022 and 2024, the system continuously monitored a road subject to blockages caused by avalanches, successfully identifying 10 events that impacted the roadway, achieving a detection rate of 100%.

The distinction of signals can be influenced by ambient noise, such as traffic or industrial vibrations, which interfere with seismic signals. In this case, advanced filtering and identification techniques are required to extract valid signals and recognize the distinguishing features of each event, such as frequency, amplitude, and propagation of seismic waves.

Recent research [74,75,76] has concentrated on classifying continuous seismic recordings by distinguishing background noise from the signal of interest. The study carried out by [76] monitored landslides in Brienz, Switzerland, in 2023 using DAS and Doppler radar, developing a semi-supervised learning model to distinguish landslide seismic signals from background noise such as vehicle and rail traffic. To improve the analysis, the cross-spectral density matrix (CSDM) was used, which made it possible to identify signal coherence over three frequency bands, improving the distinction between landslides and other events. The model detected large rockfalls with high reliability, while smaller events were less recognizable due to a lower signal-to-noise ratio. Accuracy was highest during the nights when traffic was low, while 74% of missed events were confused with traffic noise during the days. However, the system has identified hundreds of precursor events in the hours before the collapse, confirming that an increase in seismic activity can be the signal of an imminent landslide.

Xie et al. [77] evaluated the effectiveness of DAS for monitoring rockfalls compared with traditional seismometers. The experiments, conducted in a granite quarry in China, used a DAS array with optical fibers and seismometers to record seismic signals from 38 artificially released blocks. They classified seismic signals into intact rock propagation, fragmented rock propagation, and collapse-triggering propagation. The results of DAS arrays, compared with traditional seismographs, confirm their effectiveness in the detection of events.

Later, in [72], the authors tested a new method for real-time localization of rockfalls and landslides using fiber-optic distributed acoustic sensors (DASs) integrated with artificial vision techniques. In a study area in Suzhou (China), DAS cables were installed in L-shaped and parallel trenches, and artificial seismic signals were simulated by hitting a metal plate at different locations. The data collected was elaborated to determine the time of arrival of seismic signals. The method has shown greater accuracy and noise resistance than traditional techniques. The system also outperformed, in accuracy, a reference seismic node array, showing the potential of the DAS-computer vision combination for monitoring linear infrastructure in mountain areas.

Although further research is needed to fully understand its potential, the DAS’s ability to detect a wide range of events characterized by distinctive seismic signals suggests that this system could also be effectively applied to the monitoring of large-scale mass movements. This aspect opens up new perspectives for improving strategies for the mitigation of geological risks, contributing to the safety of infrastructures and communities exposed to such phenomena.

### 3.2. Subsidence Monitoring

An increasingly critical geotechnical issue stems from the intensive exploitation of underground resources such as water, oil, and gas, which can trigger ground subsidence or, in extreme cases, sudden surface collapse. Over-extraction of groundwater, particularly in unstable soils, leads to gradual surface lowering, potentially damaging infrastructure, altering runoff patterns, and heightening flood risks. Similarly, oil and gas extraction can accelerate large-scale subsidence, threaten land stability, and cause severe economic and environmental consequences. Just as with slow landslides, traditional monitoring tools, which mostly rely on topography measurements and remote sensing techniques, though useful for detecting surface movements, fall short in capturing the full depth and spatial extent of subsurface changes. In response, recent research has emphasized the use of DFOS as a more effective solution for continuous and comprehensive monitoring of subsidence and swelling phenomena.

**Figure 11:**
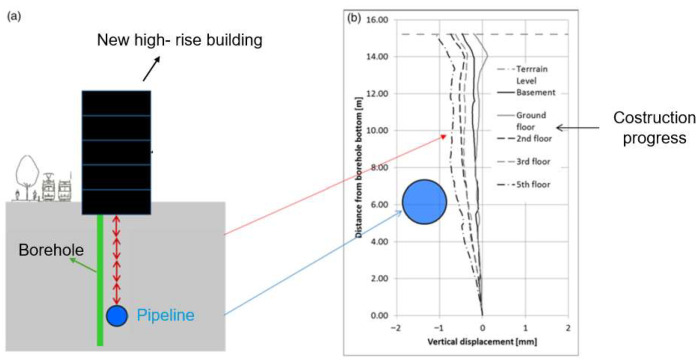
Settlement monitoring during a high-rise building construction: (**a**) schematic layout; (**b**) evolution of vertical displacement profiles (reprinted from [78] under a CC BY 4.0 license).

Study [78] deployed distributed fiber-optic sensors in 12 boreholes to monitor soil settlements around a water pipeline under a high-rise building during its construction in the city of Zurich (Figure 11). To ensure an accurate estimation of the risk associated with the accidental breakage of the pipeline during all phases of construction, the authors effectively monitored the vertical displacement profile, modeling the tolerable displacement of the pipeline using a three-dimensional finite element analysis, too.

Liu et al. [45] placed a DFOS in a 0.75 m-deep trench to monitor an area in China characterized by strong soil subsidence with the opening of ground cracks due to intensive groundwater extraction. The results compared with GPS data showed that the detected strain values were consistent, offering a much higher spatial resolution. Furthermore, by comparison with manual fracture measurements, the authors showed that variations detected by BOTDA visibly preceded the expansion of fractures, demonstrating the potential of optical fiber for rapid alert.

**Figure 12:**
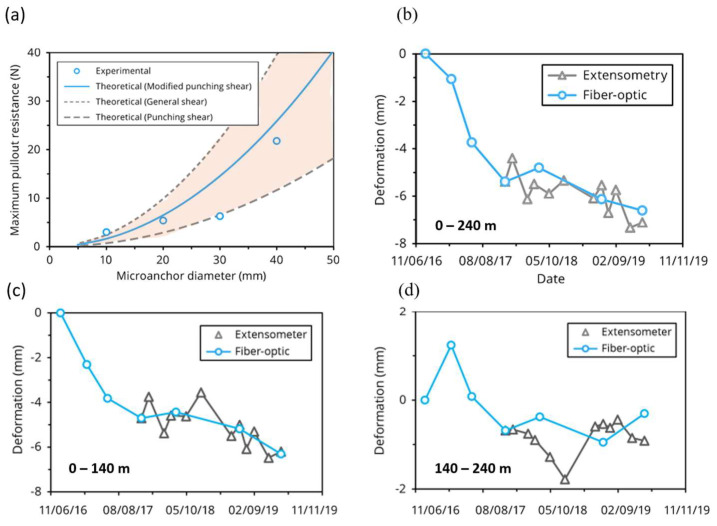
(**a**) Comparison between experimental and theoretical maximum pull-out resistances for disc-shaped micro-anchors during laboratory tests. Alongside the modified punching shear model used in this study, upper and lower bounds based on general and punching shear failure mechanisms are also shown (red zone); (**b**) comparison between cumulative extensometer and fiber-optic strain data in a 240 m-deep borehole; (**c**) comparison between cumulative extensometer and fiber-optic strain data at 0–140 m depth; (**d**) comparison between cumulative extensometer and fiber-optic strain data at 140–240 m depth (reprinted from [79] under a CC BY 4.0 license).

In a similar context, Sevillano et al. [16] applied the BOTDA technique to measure the vertical displacement patterns of a slow-moving sinkhole in Spain. They installed a fiber-optic cable in a flood-protection embankment and compared the data with high-precision leveling measurements. The results showed a strong spatial and temporal correlation, confirming the system’s reliability for monitoring subsidence in natural conditions and its ability to detect an acceleration of the sinking process in conjunction with a flood event. Zhang et al. [79] proposed micro-anchored optical cables to optimize the cable-ground coupling, studying the response of three types of micro-anchors (disc, cylinder, and spindle) fixed to the optical fiber cable by means of epoxy resin in developing distributed extensometer measurements for settlement monitoring. They developed a theory for the assessment of coupling based on that of bearing capacity in geotechnics. To check whether this theory was suitable for describing the phenomenon, the authors carried out pull-out tests by comparing measured resistance values with theoretical ones. The results showed that a fair agreement was reached (Figure 12a). Later, the authors utilized the developed approach for monitoring subsidence in the city of Yancheng, China, due to underground resource extraction. In this case, the tested DFOS sensor, equipped with micro-anchors, was installed inside a 240 m-long borehole. During more than two years of monitoring, strain data acquired with the fiber-optic system showed agreement with independent extensometer measurements (Figure 12b). In detail, for the depth range 140–240 m (Figure 12d), the differences found between the optical fiber measurements and those of strain gauges are attributable to the small magnitude of the strain values and the limited availability of reference points. Despite this, for the intervals 0–140 m (Figure 12c) and 0–240 m (Figure 12b)—which include the surface layer most subject to compression (0–20 m)—the data obtained with the two methods showed significant agreement, both in terms of trend and absolute values.

Liang et al. [80] utilized the city of Tianjin in China, an area with historical subsidence up to 3.25 m, as a case study for testing an innovative methodology. They drilled a 100 m-deep monitoring well (G06), equipping it with a fixed-point optical cable and monitoring soil subsidence by using BOTDR. The results showed uneven subsidence, with the shallower marine layers (3.4–18.4 m) accounting for 61.5% of the total settlement (32 mm). Integrating DFOS data with a proposed Logistic Model allowed the authors to predict a cumulative settlement of 56 mm by December 2019, with an estimated limit of 96 mm and stabilization projected by 2050. The main layers (0–3.4 m and 18.4–38.4 m) with high subsidence rates (25.0 mm/year) were identified, requiring attention. Validation with extensometers and GPS/InSAR confirmed DFOS reliability, demonstrating their potential as a crucial predictive and decision-making tool for subsidence risk management.

Furthermore, a critical experimental validation of DFOS effectiveness in detecting settlements was provided by the airbag inflation/deflation test [81]. This controlled experiment aimed to demonstrate the capability of DFOS to precisely measure both uplift and subsidence. The setup involved installing an optical fiber cable in a shallow trench, with airbags strategically placed underneath a pre-excavated space. By controlled inflation and deflation of these airbags, measurable and reversible ground deformations were induced, allowing for a direct correlation between mechanical action and sensor response. The key advantage of this methodology lies in its unique ability to simulate and monitor both upward and downward vertical movements with high sensitivity and resolution, which is essential for comprehensive ground dynamics analysis where both subsidence and swelling phenomena may occur. This bidirectional validation significantly enhances confidence in applying DFOS for reliable geomechanical monitoring in various field conditions. 

Yeskoo and Soga [82] introduced an innovative CPT-based installation method for distributed fiber optic sensing (DFOS) to monitor vertical ground settlements at Treasure Island, San Francisco. The fiber-optic cable, pre-tensioned and anchored to a sacrificial cone, was inserted using CPT rods, reducing both installation time and cost compared with traditional borehole methods. The borehole was then backfilled with a cement-bentonite grout to ensure mechanical coupling with the surrounding soft clay (Young Bay Mud). Monitoring was performed using a BOTDR analyzer, providing continuous strain profiles with a 0.75-m spatial resolution. Results showed strong agreement with traditional topographic surveys for strains below 2000 με, effectively capturing differential settlements and localized strain concentrations. Fiber decoupling at higher strains remains a constraint, indicating the need for improved anchoring systems for larger settlements.

### 3.3. Earthquake Monitoring

**Figure 13:**
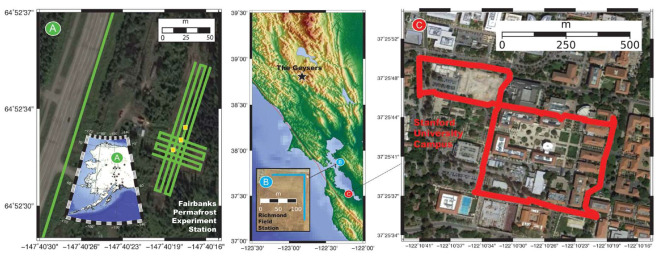
Color-coded maps of the fiber-optic arrays used for the experiments at (**A**) the Fairbanks Permafrost Experiment Station, AK, (**B**) Richmond Field Station, CA, and (**C**) Stanford University, CA. The green, cyan and red lines indicate fiber cables directly installed in shallow trenches or conduits [84].

The attempt to use fiber optics distributed sensors for seismic waves detection and monitoring dates to 2013 [83], when purposely deployed optical fiber cables, with different installation procedures (cemented, fluid coupled, etc.), were tested with artificial surface seismic sources (such as vibroseis trucks and weight-drop technique). Comparison with geophones data proved the substantial effectiveness of the approach. On the other hand, the possibility of exploiting the optical fiber telecommunication networks to detect earthquakes was firstly devised by a group of researchers from the United States [84]. To demonstrate the effectiveness of the approach, they performed three experiments using telecommunication fiber-optic cables as sensor arrays, enabling meter-scale recording over tens of kilometers of linear fiber length. Taking advantage of the DAS technology, they were able to detect seismic waves at three different locations: the Fairbanks Permafrost Experiment Station in Alaska (USA), the Richmond Field Station in California (USA), and Stanford University, California (USA) (see Figure 13).

The DAS records were compared with conventional inertial seismometers, and comparable estimates of ground motion, including body wave and surface wave travel time, peak ground acceleration and coda envelope shape, were found. The array nature of DAS, as opposed to a single seismic point sensor, enables identification of the back azimuth and slowness of arriving earthquake energy, as well. Furthermore, one fiber array installed inside a conventional telecommunications conduit showed how existing fiber-optic networks might also be exploited, in spite of the reduced signal-to-noise ratio due to the lack of direct ground coupling.

**Figure 14:**
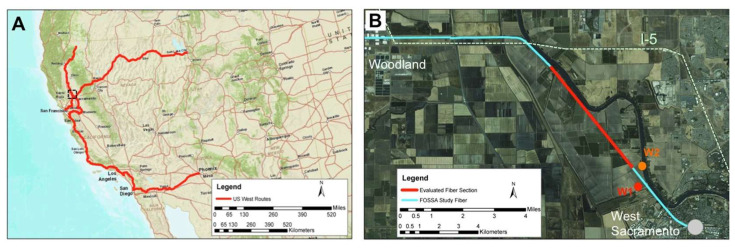
(**A**) The regional network within California and western Nevada; (**B**) the subsection of the network used in the study. The red segment in (**B**) is the area of focus for ambient noise analysis (reprinted from [85] under a CC BY 4.0 license).

Subsequently, another group of researchers at the U.S. Department of Energy’s Lawrence Berkeley National Laboratory (California, USA) performed tests on a conventional fiber-optic link, also showing how to cope with the georeferentiation of the cable and the environmental noise. They turned parts of a 13,000-mile-long testbed of “dark fiber,” unused fiber-optic cable, owned by the DOE Energy Sciences Network (ESnet), into a highly sensitive seismic activity sensor that could potentially augment the performance of earthquake early warning systems [85]. The seismic dataset they presented was recorded between 28 July 2017, and 18 January 2018, using a DAS interrogation unit (IU; Silixa iDAS, Elstree, UK) installed in a telecommunication Point-of-Presence (PoP) facility in West Sacramento (see Figure 14). Ambient seismic noise was recorded using the DAS IU at 500 Hz sampling with a spatial sampling of 2 m while the gauge length was 10 m. While the surface geometry of the dark fiber network was known before deployment, the mapping to linear fiber location was established by sequential impact tests at surface locations surveyed with high-accuracy differential GPS. This is fundamental due to the common practice of including spools of loose cable during telecom installation, so the actual length of the fiber does not map to a precise position on the surface. By establishing true differential GPS coordinates for the test impact locations, it was possible to compensate for the extra fiber length effects when mapping back to the previously surveyed deployment geometry. Then, DAS signal strength, amplitude, and periodicity, as well as the noise characteristics along the array, were examined. Dominant noise features, including several regional highways, diffuse urban noise, and energy from local railroad activity, were identified. This illustrates the potential heterogeneity of signal quality across the existing telecom network. Interestingly enough, the dark fibers running near strong sources of environmental noise (such as railway lines) can exploit this noise as a source of seismic energy for both P- as well as S-wave imaging and detect hydrologic variations and other environmental parameters of geotechnical/geological interest.

The same fibers were used to record two earthquakes of similar magnitude that occurred on 18 January 2018, at the geysers and on 4 January 2018, at Berkley, respectively.

**Figure 15:**
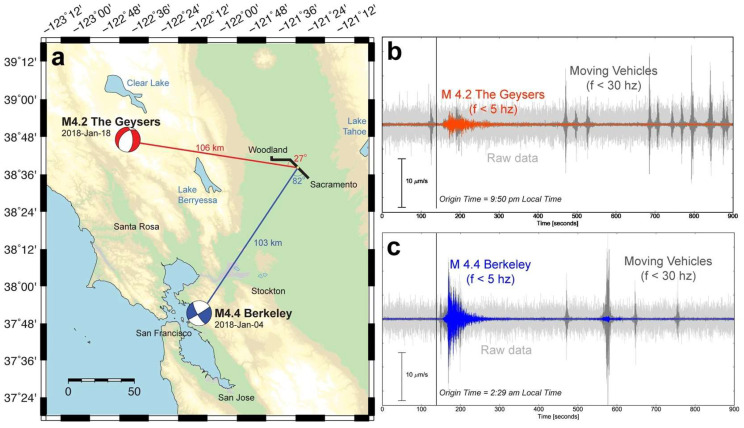
(**a**) Locations and focal mechanisms of the 18 January 2018 geysers (magnitude 4.2, red line) and 4 January 2018 Berkeley (magnitude 4.4, blue line) earthquakes, which occurred approximately 100 km from the Sacramento Dark Fiber DAS array (black line). (**b**,**c**) Raw (grey lines) and low-pass filtered (red and blue lines) DAS strain-rate waveforms for these events averaged over 100 m (reprinted from [85] under a CC BY 4.0 license).

Figure 15 reports the earthquake’s location along with the strain recordings (raw and low-pass filtered). The low-pass filtering clearly allows for discriminating between the earthquake-generated seismic waves and those generated by the passing vehicles.

From Figure 15, it appears that the earthquake records look very different despite having been generated in similar-sized ruptures and traveling similar distances to Sacramento. This may be due to source rupture depth differences (z = 2.4 km for the geysers, z = 12 km for Berkeley), and/or the major differences in geologic structure along the seismic waves’ path and the orientation with respect to the fiber axis, as well.

Of course, the optical fiber cable structure and installation method can also significantly affect the recording and detection sensitivity.

Moving further on toward the possibility of exploiting optical fiber networks for earthquake monitoring and analysis, it should be kept in mind that the traditional seismometer networks must face some definite challenges:

- The need for stringent (millisecond) synchronization between sensors, typically requiring a global positioning system (GPS) clock (only available on the surface);
- The reliability and cost of data transmission in real time;
- The individual power supply, battery life, and maintenance of each sensor.

On the other hand, when using distributed optical fiber sensors, the individual points within the fiber are intrinsically synchronized because all points are interrogated with the same unit. Distributed optical fiber sensors represent an inexpensive solution because of their long lifespan and their intrinsic low cost per monitoring point, when dealing with long distances, also considering that the huge amount of available data is transmitted via the fiber itself. Specifically, the cost of the sensor itself (a standard optical fiber) can be as low as a fraction of a dollar per meter, i.e., orders of magnitude less than point-based technologies such as strain gages and fiber Bragg gratings. Furthermore, the ability to perform remote measurements allows the interrogation unit to be kept in a safe place away from harsh or hardly accessible locations. This is especially true for subsea monitoring, as it prevents expensive maintenance actions or the need for a co-located power supply.

The availability of a huge amount of data requires, of course, signal processing approaches based on Artificial Intelligence, acoustic beamforming, and multidimensional processing.

**Figure 16:**
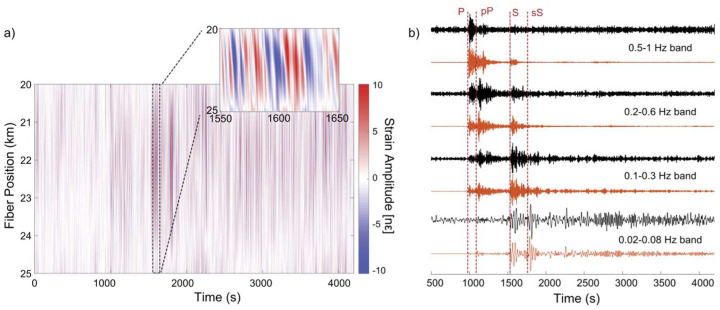
(**a**) Strain recordings vs time and length after 2D linear filtering. Only seismic waves propagating from the west to the east have been detected. (**b**) Comparison of DAS traces (black lines) and those obtained by using the nearby seismometer (orange lines) at the W–E direction, filtered to various bands between 0.02 Hz and 1 Hz and normalized to particle velocity (reprinted from [86] under a CC BY 4.0 license).

Interesting results have been reported in [86], where an M8.2 earthquake that occurred in Fiji was analyzed with a telecommunication fiber located in Pasadena (CA, USA) at more than 9000 km from the epicenter, exploiting a chirped-pulse DAS interrogator unit. The raw data were affected by a huge amount of environmental noise, but after proper filtering and using suitable denoising algorithms, the comparison with data collected by a nearby broadband seismometer showed a good agreement (see Figure 16).

A major difficulty of DAS in seismology is its one-directional sensitivity, as the fibers only detect axial strain. A solution based on helically wound optical fibers has already been proven [87], offering 3D sensing of seismic waves, but, of course, it requires custom fiber cable installation and cannot be exploited with existing telecom links.

**Figure 17:**
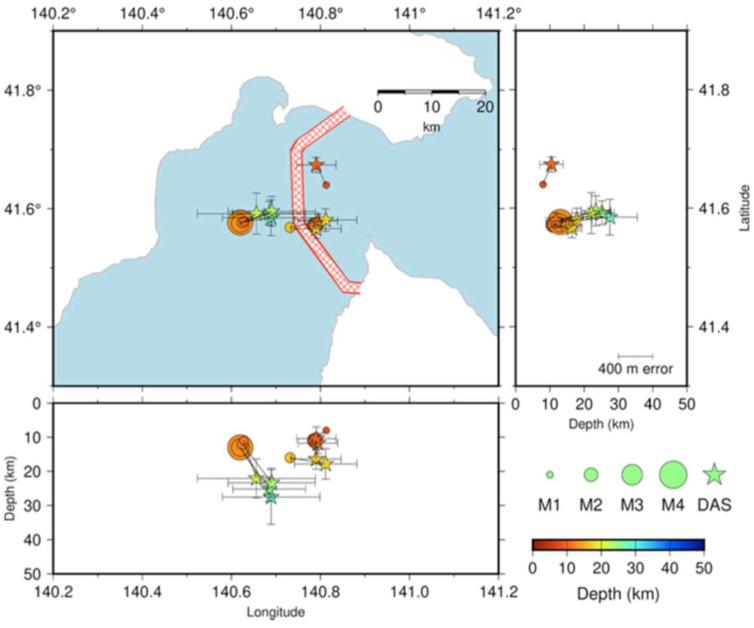
Earthquake locations determined by DAS data (stars) and JMA (circles). Black lines connect the corresponding events. The fiber-optic cable is in the red-dashed area (reprinted from [88] under a CC BY 4.0 license).

The possibility of exploiting offshore fiber-optic cables to locate earthquakes near the cable and derive the empirical relationship between the magnitude and DAS S-wave strain rate amplitude was demonstrated by Satoru Baba et al. [88] through measurements carried out over a 4-month period using a cable deployed in the Tsugaru Strait. In this measurement campaign, some earthquakes with magnitudes smaller than one or not listed in the earthquake catalog by the Japan Meteorological Agency (JMA) were observed. For all events, the difference between the epicenters located using DAS data and those by the JMA catalog was less than 0.1° (see Figure 17).

Moving toward more sophisticated processing of DAS data, it is worth mentioning the work by Strumia et al. [89], where the authors presented a new formulation for far-field strain radiation from seismic events, leading to a direct interpretation of data to retrieve source properties (seismic moment and source size), via spectral modeling. The technique has been successfully validated on real data recorded in two different tectonic environments, the Chilean margin and the southern Apennines, in Italy. In the first area (Chile), the DAS interrogator was connected to a submarine fiber-optic telecom cable, while in the second one (Italy), an L-shaped cable was purposely deployed in a dry lakebed.

**Figure 18:**
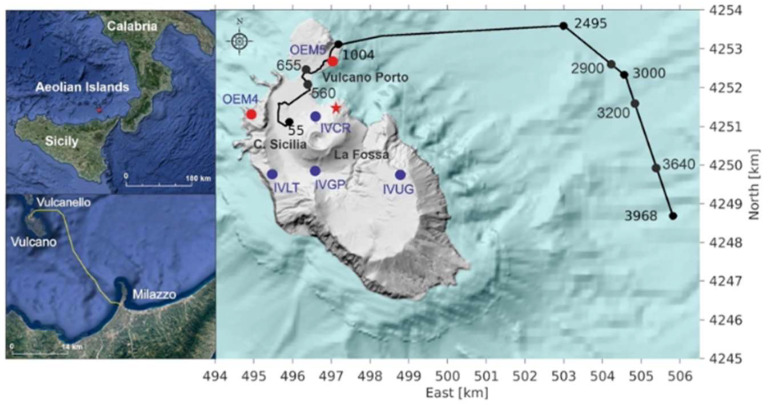
The fiber-optic cable path from Vulcano Island to Sicily. The blue dots and the red dots represent the permanent and mobile broadband seismic stations operating on land at Vulcano Island. The red star indicates the location of the source of the VLP events. (reprinted from [90] under a CC BY 4.0 license).

An interesting monitoring campaign has been carried out by the Italian National Institute of Geophysics and Volcanology (INGV). In their work [90], the authors present findings from a month-long acquisition at Vulcano Island in Sicily, Italy, an area affected by diffuse signals of volcanic activity. They used a telecom fiber-optic cable link, running partly on-shore and partly offshore between Vulcano Island and Sicily (Figure 17) and a DAS interrogator, namely iDAS^®^ from Silixa. A fixed gauge length of 10 m was applied to record the strain rate data with a sampling frequency of 1 kHz and a spatial sampling of 4 m. During the acquisition period, they found 1488 events with a great variety of waveforms composed of two main frequency bands (from 0.1 to 0.2 Hz and from 3 to 5 Hz) with various relative amplitudes. The results were compared with seismic records from the land-based monitoring system managed by INGV-OE (Figure 18).

Even if the geometry of the fiber cable and its coupling with the ground are not optimal to sense low-frequency volcano seismicity, signatures of the seismic events are very well-recognized in the DAS signals, and also in the submarine section of the cable. Due to the very nature of the telecommunication cable, i.e., not bound to the ground but just “floating” inside the cable conduit, very low amplitude signals at the resolution limit of the instrument are detected. However, the weakness of the signal is balanced by the higher spatial density of the observations that allows for robust detection with properly adapted Machine Learning (ML) approaches for image processing. In fact, the detection performance of the Convolutional Neural Network (CNN) on the continuous data suggests that, after initial training, the ML approach could be efficiently used for near real-time monitoring.

The exploitation of optical fiber distributed acoustic sensing for seismic waves detection is gaining more and more attention, also due to applications different from the mere earthquake monitoring, such as microseismicity monitoring for energy production, carbon sequestration and near-surface imaging [91] and the different performances, exhibiting unique characteristics often better, in comparison to other fiber-optic-based techniques [92,93]. Offshore earthquakes and tsunami monitoring are surely extremely interesting applications, also due to the lack of offshore traditional seismic stations and the need for early warning systems [94,95,96]. On the other hand, a great research effort is still mandatory to fully exploit all the potentialities of this technology, addressing problems such as directional sensitivity limitations, calibration inconsistencies, saturation effects, and vast data volumes handling, also taking advantage of advanced Machine Learning techniques [97,98].

## 4. Discussion

This review of the state-of-the-art of DFOS applications for some of the most hazardous geohazards highlighted two main fields of applications, as reported in Table 2, which summarizes the main applications discussed, synthetically illustrating the limitations, advantages, and prospects of DFOS applications in geohazard contexts. The one based on well-established BOTDA/R, BOFDA, and OFDR interrogation techniques, whose methods and data processing are clearly defined, making their use accessible even to non-experts, and the one based on DAS technology, for which the interpretation of the acquired signals still requires expert judgment, eventually helped by ML algorithms. This makes the latter technology not yet ready for wide diffusion. Specifically, Brillouin-based techniques are especially suited for static and quasi-static strain measurements over long distances (several tens of km), while the OFDR method only works over short distances (tens of meters); therefore, it appears more suited to strain monitoring in environmentally controlled, laboratory-scale experiments [40]. On the other hand, distributed acoustic sensors are capable of long-range measurements (up to tens of km), but have a limited spatial resolution (a few meters, typically). Their high sensitivity and acquisition rate make them ideal for detecting vibrations propagating into soil, such as those produced by earthquakes or fast landslides. For even longer ranges (hundreds of km or even thousands of km), interferometric or polarimetric methods have emerged in the last few years (see, e.g., [99]). In these methods, the impact of vibration on the phase and/or state-of-polarization of the light wave propagating over the cable is detected by monitoring the properties of the transmitted light, rather than its backscattering. However, these methods provide a relatively poor spatial resolution (in the order of hundreds of meters), which is typically obtained by cross-correlating the time-of-arrival of the perturbation at the two opposite ends of two parallel fiber cables.

The consolidated and widely adopted interrogation techniques (BOTDA/R and OFDR) have undergone extensive experimentation, which has further reinforced the robustness of their data interpretation methods, proving their validated effectiveness in diverse contexts. Despite the significant advancements made during the last decade, several challenges persist:

- The high cost of commercial acquisition units restricts the feasibility of extensive real-time experimentation, primarily limiting the use of these instruments to research institutions. In this regard, a step change in terms of cost, size, and energy consumption of interrogation units is expected to be enabled, in the next few years, by silicon photonics technologies. The impact of such technologies can already be observed in the FBG interrogator market (see, e.g., [100]), but an extension to DFOS is expected soon.
- The lack of standardization in sensor packaging and installation procedures presents a notable hurdle. Currently, each experiment uses customized solutions for sensor protection and fastening, which complicates the comparability and scalability of applications. Further research is essential to identify the most suitable bonding/protective materials and embedding techniques for each specific implementation.
- Field setups for the detection of fast landslides are still few, avoiding a clear evaluation of their feasibility in this field of application.

On the other hand, it is important to summarize the advantages and the critical aspects of the approach based on DAS technology:

- Exploiting dark fibers in already deployed telecom fiber-optic cables surely represents an advantage, because it allows monitoring large areas, and especially the seafloor, where no conventional seismic stations are present. Exploiting non-purposefully deployed fiber-optic cables, i.e., cables running inside cable ducts and not tied to the ground, whose path and real constraint conditions (boundary conditions) are not known, represents an important limitation. In fact, a scarce mechanical coupling between the cable and the ground can lead to poor results [101].
- The ability to detect only strains applied along the fiber axis also represents a significant limitation [102].
- The environmental and anthropogenic noise can largely affect the ability of the sensors to detect seismic events in a reliable way.
- Machine Learning algorithms are always required to extract the significant features from a huge amount of available data [103].
- Due to the unconstrained nature of the telecom fibers, quantitative information about the actual amplitude of the seismic waves cannot be extracted, but only the differences between different sections of the fiber can be made available.

The spectral features along with the direction and time of arrival of the seismic waves can be extracted, provided that at least the path of the fiber cable is known [104].

When not using constrained fibers specifically installed to perform DAS measurements, the possibility of obtaining quantitative data is still not realistic for setting up of monitoring systems run realistic, whereas the idea of exploiting already deployed telecom optical fiber networks represents, probably, the initial step in showing that fiber optic sensing can be utilized in real-time to assist in monitoring seismic activity, especially to cover areas where no seismic stations are installed. Of course, whenever and wherever optical fiber cables can be installed specifically for sensing purposes [105], with well-known paths and boundary conditions, the achievable results from DAS data analyses, via ML algorithms, could represent a promising tool to increase the knowledge of seismic phenomena and set up early warning systems covering large areas.

DFOS integration with IoT technology to enhance the efficiency of monitoring and early warning systems represents an emerging and promising field of study, but the current literature still lacks significant experiments or large-scale implementations. Although IoT systems are well-suited for developing integrated early warning networks, meaningful applications in the specific domain of geohazards remain limited at this stage. Some emerging trends, such as measurement automation, Artificial Intelligence (AI), and photonic integration, are instead revolutionizing DFOS applications. Regarding measurement automation, industrial applications are increasingly relying on integrating one or more DFOSs with Supervisory Control and Data Acquisition (SCADA) and Geographic Information Systems (GISs), by which data are collected and accessed from a remote location. DFOS produces a vast amount of data, making its integration in a SCADA environment more challenging with respect to conventional discrete sensors. This is particularly relevant for distributed acoustic sensors (DASs), for which some edge processing is usually required to reduce traffic volume and latency. System integration makes possible the cooperation of DFOS with other sensors, as well as alarm management and actionable intelligence with specific thresholds based, e.g., on localized strain or temperature spots, or specific acoustic patterns. The use of Machine Learning (ML) or Artificial Intelligence (AI) techniques may be highly beneficial, as they assist in identifying any hazard condition from DFOS data in a fully automatic and robust manner. Major developments have been seen in the last decade, related to the use of ML/AI techniques to automatically detect threats [106] or enhance sensor capabilities through data denoising or multiparameter sensing [107]. In distributed acoustic sensing, ML/AI techniques are increasingly used to distinguish between routine events like vehicular traffic or digging machinery from vibrations produced, e.g., by geohazard events. However, the lack of large fiber-optic sensing datasets labeled with geohazard events complicates the training of robust early warning ML models, unless proper data generation strategies are developed to simulate DFOS data linkable to acoustic events of interest. Finally, photonic integration is expected to be a game changer for DFOS technology in the next few years, because moving from discrete components to silicon photonic chips shows great promise in size, weight, power and cost reduction of DFOS interrogators [108]. These are all factors that may play an important role in increasing the overall acceptance and sustained usage of DFOS technologies.

## 5. Conclusions

Distributed optical fiber sensing (DFOS) technologies have proven highly effective for geohazard monitoring, with Brillouin- and OFDR-based methods offering robust and validated strain measurements, whereas DAS shows unique potential for seismic and vibration monitoring over large distances. However, widespread adoption is still limited by high equipment costs, lack of standardized sensor packaging, and challenges in field deployment, particularly for fast landslide detection. DAS technology offers the advantage of exploiting existing telecom fibers to extend monitoring coverage, especially where no standard seismic stations are installed, but it faces significant limitations in spatial resolution, quantitative accuracy, and susceptibility to noise, requiring advanced Machine Learning algorithms for better data interpretation. Overall, while consolidated methods are reliable and mature, DAS technology represents a promising but still developing approach, whose full potential could be unlocked through purpose-deployed fibers, improved interrogation technologies, and standardized practices, ultimately enabling large-scale, real-time early warning systems.

## Figures and Tables

**Table 1:** DFOS technologies and their performance.

Sensing Technology	Sensing Parameters	Accuracy	Sensing Range	Spatial Resolution	Advantages	Limitations
BOTDR	Strain Temperature	±20 µε ±1.0 °C	≈100 km	≈1 m	Good sensitivity, single-ended measurements	High equipment cost, limited spatial resolution
High equipment cost, limited resolution for long-distance BOTDA	Strain Temperature	±20 µε ±1.0 °C	≈100 km	10 cm–1 m *	Good sensitivity, high spatial resolution over medium distances	High equipment cost, limited spatial resolution over long distances
BOFDA	Strain Temperature	±20 µε ±1.0 °C	80 km	1 cm–1 m *	Very high spatial resolution	Costly equipment, complex system setup
OFDR	Strain Temperature	±2 µε ±0.1 °C	70 m	≈1 mm *	Good sensitivity, ultra-high spatial resolution	High equipment cost, limited sensing distance
Φ-OTDR	Strain Temperature Vibration	±10 nε	100 km	1–10 m *	High sensitivity, fast acquisition rate	High equipment cost, complex data processing, limited spatial resolution

* Varies with sensing distance.

**Table 2:** Overview of DFOS techniques for geohazard monitoring.

DFOS Method	Spatial Resolution/Accuracy	Application	Achievement	Advantages	Disadvantages	Reference
BOTDA	0.05 m to 1 m/7.5 µε to 20 µε	Trench in roads to locate sliding mass boundaries	Qualitative agreement with traditional instruments	Distributed strain monitoring with spatial resolution higher than traditional instruments	Cable protection, installation procedure, and data interpretation methods	[52,53,55,65,66] * [23,71] ** [16,45,78,79,80] ***
		Vertical extensometer for monitoring subsidence				
		DFOS sensors with micro-anchors: laboratory and field tests				
		Monitoring of soil subsidence and soil crack formation	Quantitative measurements in agreement with traditional instruments			
		Laboratory test on DFOS-inclinometer				
		In-site monitoring of fast rain-induced landslide				
		Study of a slow-moving sinkhole				
BOFDA	0.2 m to 0.5 m/20 µε	Monitoring of rain-induced landslides in a physical model				
		Extenso-Inclinometer for monitoring of slow landslide		Standardization of the fiber optic installation process and 3D detection of slope deformation	Need for specialist skills for data interpretation; high cost of the control unit	[58] *
BOTDR	0.75 m to 1.0 m	Monitoring of the Mejiagou landslide	Identification of the deformation phenomenon.	Ability to detect and interpret complex strain distributions; provides detailed insights into landslide mechanics and evolution	Installation procedure and data interpretation methods; need for a traditional instrument to interpret strain distributions	[53,66] * [80] ***
		Subsidence monitoring				
		CPT-based installation of fiber optics to monitor vertical displacements				
OTDR	0.25 m to 0.5 cm	Laboratory validation of a 36 mm-diameter rod integrating a fiber optic sensing (FOS) system and on-site tests	The progressive deformation behavior of the slope was accurately captured	Enables potential sensor multiplexing for deep landslide monitoring.	The system remains quasi-distributed, and field testing is required to validate its effectiveness	[55] *
OFDR	10 mm/not specified	Monitoring of rain-induced surface landslides in a large-scale physical model using FO cable anchored with geogrids	Identification of deformation phases	High spatial resolution, allowing instability precursors to be detected in unprecedented detail	Sensitivity to installation conditions; limitations of the physical model; costs and skills	[40] ** [81] ***
		Optical fiber installed in a surface trench; airbag inflation/deflation test to simulate deformations				
DAS	4 m/µε	A 925 m-long fiber-optic cable buried 10 cm deep; monitoring during a three-day rainy event	Detection of hidden deformation processes, classification of seismic signals	High sensitivity; ability to detect dynamic phenomena in real time; ability to detect precursor events	Need to optimize cable geometry to detect deeper, multidirectional deformations; poor mechanical coupling between cable and ground; limited ability to detect small events	[67] * [73,76,77] **
		Laboratory simulation of artificial seismic signals				
		Use of dark fibers for avalanche monitoring				
		Landslide monitoring				
DAS	1 m to 100 m	Existing telecom fibers	Good agreement with traditional seismometers	Large area monitoring, subsea monitoring, no need for co-located power supply	Detection of strain components along the fiber path only, low signal-to-noise ratio, ML algorithms required for data analysis	[83,84,85,86,88,92,93,94] ****
		Custom-deployed fibers		3D strain detection exploiting helically wound fiber cables		[87] ****
				L-shaped fiber cable: strain in two orthogonal directions		[89] ****

* Slow landslides, ** Fast landslides, *** Subsidence, **** Earthquakes.

## Data Availability

Data are contained within the article.

## Abbreviations

The following abbreviations are used in this manuscript:

BOTDA	Brillouin Optical Time-Domain Analysis
BOFDA	Brillouin Optical Frequency-Domain Analysis
BOTDR	Brillouin Optical Time-Domain Reflectometry
ROTDR	Raman Optical Time-Domain Reflectometry
OFDR	Optical Frequency-Domain Reflectometry
OTDR	Optical Time-Domain Reflectometry
Φ-OTDR	Phase-sensitive Optical Time-Domain Reflectometry
